# Odds and Ends

**Published:** 1869-11

**Authors:** John Blake


### ODDS AND ENDS.

*Gentlemen of the Mad River Valley Dental Association:*—
It is not my intention to write an essay, but merely to have (through the mediumship of my pen) a little social chat with you, on a few odds and ends of subjects, that may be interesting to the society.

I never fully appreciated our society meetings until after coming to the "far west," where, cut off from all professional association, I have to fall back entirely on my own resources. But, gentlemen, I am going too fast. Once a month a society (not named yet) meets in my office. It consists of the REGISTER, COSMOS, DENTAL O & L. and myself. There is always a full attendance, and the meetings very interesting and instructive. The convocations last a month, and adjourn *ad interim* for clinics, and for refreshment and sleep.

Even in this new country we are not free from those pests of society, the *traveling quacks.* There are no less than six of them within ten or fifteen miles of this place.

For patrons, I have all classes and nationalities, with a very fair sprinkling of refined, educated people, who appreciate what God has given them, and use every means in their power to preserve their natural teeth. It is a pleasure to work for the latter class, and I trust the time is not far distant, when we will have every inhabitant of our glorious country dentally educated to the importance of caring for their natural organs, instead of allowing them to be "yanked out" by impostors, to be replaced with those, which, at the best, are very poor substitutes for what nature has given us. Start the "Dental Tracts" gentlemen. It is a splendid idea, and if some one does not make a move in this direction soon, I shall have to commence it, by writing short articles for our home papers.

There has been a number of accidents reported lately, caused by the explosion of vulcanizers, and it occurs to me that they may be occasioned by not getting the collar securely screwed on the boiler. (I have reference to the Hay's machine.) There is more danger to be apprehended when using a new thick packing, which will only let one or two of the screw threads of the collar catch. This is the weak part of the machine, and those using these vulcanizers would do well to observe the following directions in putting them together.

Turn you collar on the boiler as far as possible, and then screw the nuts on the top of the collar well down to settle the packing, now loosen the nuts so that they do not protrude through the lower part of the collar, and you will find the collar will turn down enough to make one or two more of the threads catch. Repeat this two or three times if necessary, and you will make your vulcanizer much safer.

We have all, I suppose, at some time or other, experienced trouble in making full lower dentures satisfactory to ourselves and to the patient, in those cases where there is scarcely any ridge, and the muscles so prominent as to require something weightier than vulcanite to keep the plate to its place—where cheoplastic is too burdensome, and the patient not able to pay for continuous gum work. To remedy these difficulties, I have successfully used a combination of cheoplastic and vulcanite, which makes a very neat job, and gives the operator the advantage of being able to regulate the plate to any desired weight, by making the cheoplastic heavier or lighter. In making this work, I first cast a cheoplastic base, taking care to make it heavy enough (for it is easily made lighter). Then with an old excavator, sharpened like a graver, I gouge the upper surface of the plate in opposite directions till it has somewhat the appearance of a rasp. These gouges are all that is necessary to hold the two bases together. Now put wax on the plate and take the bite and grind and arrange your teeth as for any vulcanite case.

In putting in the flask, only immerse the metal to the top of the rim in the plaster in the lower part of the flask, and then proceed as with an ordinary vulcanite case. It is very little trouble to make these cheoplastic bases, and give you the satisfaction of knowing whether your plate will fit before your work is finished.

And now, gentlemen, if anything I have written proves of the least benefit or has been in any way interesting to you, I shall feel amply repaid for my effort. Hoping at some future time to meet with you again, I remain

Yours fraternally,

JOHN BLAKE.

